# Prognostic value of right atrial strain derived from cardiovascular magnetic resonance in non-ischemic dilated cardiomyopathy

**DOI:** 10.1186/s12968-022-00894-w

**Published:** 2022-11-10

**Authors:** Yangjie Li, Jiajun Guo, Weihao Li, Yuanwei Xu, Ke Wan, Ziqian Xu, Yanjie Zhu, Yuchi Han, Jiayu Sun, Yucheng Chen

**Affiliations:** 1 Department of Cardiology, West China Hospital, Sichuan University, Chengdu, 610041 Sichuan China; 2 Department of Geriatrics, West China Hospital, Sichuan University, Chengdu, 610041 Sichuan China; 3 Paul C. Lauterbur Research Centre for Biomedical Imaging, Shenzhen Institutes of Advanced Technology, Guangdong, 518055 China; 4 Wexner Medical Center, College of Medicine, The Ohio State University, Columbus, OH 43210 USA; 5 Department of Radiology, West China Hospital, Sichuan University, Chengdu, 610041 Sichuan China

**Keywords:** Right atrium, Cardiovascular magnetic resonance, Dilated cardiomyopathy, Prognosis

## Abstract

**Background:**

The value of right atrial (RA) function in cardiovascular diseases is currently limited. This study was to explore the prognostic value of RA strain derived from fast long axis method by cardiovascular magnetic resonance (CMR) in patients with non-ischemic dilated cardiomyopathy (DCM).

**Methods:**

We prospectively enrolled patients with DCM who underwent CMR from June 2012 to March 2019 and 120 age- and sex-matched healthy subjects. Fast long-axis strain method was performed to assess the RA phasic function including RA reservoir strain, conduit strain, and booster strain. The predefined primary endpoint was all-cause mortality. The composite heart failure (HF) endpoint included HF death, HF readmission, and heart transplantation. Cox regression analysis and Kaplan–Meier survival curve were performed to describe the association between RA strain and outcomes.

**Results:**

A total of 624 patients (444 men, mean 48 years) were studied. After a median follow-up of 32.5 months, 116 patients (18.6%) experienced all-cause mortality and 205 patients (32.9%) reached composite HF endpoint. RA function was impaired in DCM patients compared with healthy subjects (all *P* < 0.001). After adjustment for covariates, RA reservoir strain [hazard ratio (HR) (per 5% decrease) 1.19, 95% confidence interval (CI) 1.03–1.37, *P* = 0.022] and conduit strain [HR (per 5% decrease) 1.37, 95% CI 1.03–1.84, *P* = 0.033] were independent predictors of all-cause mortality. Moreover, RA strain added incremental prognostic value for the prediction of adverse cardiac events over baseline clinical and CMR predictors (all *P* < 0.05).

**Conclusion:**

RA strain by fast long-axis analysis is independently associated with adverse clinical outcomes in patients with DCM.

*Trial registration*: Trial registration number: ChiCTR1800017058; Date of registration: 2018-07-10 (Retrospective registration); URL: https://www.clinicaltrials.gov

**Supplementary Information:**

The online version contains supplementary material available at 10.1186/s12968-022-00894-w.

## Introduction

Non-ischemic dilated cardiomyopathy (DCM) is a heart muscle disease characterized by ventricular dilation and contractile dysfunction in the absence of coronary artery disease and abnormal loading condition. Despite the advances in the management of DCM patients, the prognosis remains poor [1, 2]. The enlarged size and impaired function of left ventricle (LV), left atrium (LA) and right ventricle (RV) are associated with worse clinical outcomes [3–6]. However, the right atrial (RA) function and its clinical implication in DCM patients has not been studied.

The RA is less studied in many cardiovascular conditions and has been deemed as a neglected chamber [7]. Its electromechanical function and endocrine regulation are pivotal for cardiac function. Like the LA, RA phasic function can be divided into three parts during the cardiac cycle: (1) reservoir function for collecting blood from inferior and superior veins during atrial diastole and ventricular systole; (2) conduit function, a passive filling phase during early ventricular diastole; and (3) booster function by atrial actively contracting during late ventricular diastole [8]. Compared with volumetric measure derived cardiac function, strain may be a more sensitive index in describing phasic performance. The development of cardiovascular magnetic resonance (CMR) imaging techniques made measuring strain parameters reliable and reproducible. A fast long-axis strain method has been proposed recently to quantifying myocardial deformation [9, 10]. Compared with the traditional feature-tracking, the fast method demonstrated superior reliability and reproducibility, with a great reduction of analysis time [9]. LA strain derived from this method showed independent prognostic value in ST-segment elevation myocardial infarction and hypertrophic cardiomyopathy [11, 12]. Leng et al*.* applied similar method to RA in patients with pulmonary artery hypertension and demonstrated that impaired RA strain reflected RV decompensation and predicted adverse cardiac events [13]. The impact of RA strain on prognosis in patients with DCM has not been explored. We hypothesized that patients with DCM suffered from RA functional impairment, especially among patients with adverse clinical outcomes. Thus, the purpose of this study is to assess the prognostic importance of RA strain by fast long-axis method in patients with DCM and evaluate whether RA strain can add incremental prognostic value over traditional risk factors.

## Method

### Study population

Patients with DCM who underwent CMR imaging from June 2012 to March 2019 were prospectively enrolled in this study. DCM was diagnosed in accordance with the classification of cardiomyopathies from the European Society of Cardiology Working Group on Myocardial and Pericardial Diseases [14]. Exclusion criteria included significant coronary artery disease, defined as > 50% diameter stenosis of epicardial coronary artery on x-ray coronary angiography or coronary computed tomography (CT), a history of myocardial infarction or infarct patterns of late gadolinium enhancement (LGE); primary heart valvular disease; congenital heart disease; arrhythmia related cardiomyopathy; infiltrative heart disease; constrictive cardiomyopathy; acute myocarditis; and peripartum cardiomyopathy. In addition, patients with excessive acholic consumption, contradiction to CMR, atrial fibrillation or significant arrhythmia during CMR, and poor image quality were also excluded in this study. 120 sex- and age-matched healthy volunteers were selected from our database [15]. This study was approved by the institutional ethics of West China Hospital of Sichuan University. All subjects provided a written informed consent.

### CMR protocol

CMR was performed on a 3T CMR scanner (MAGNETOM, Tim Trio system; Siemens Healthineers, Erlangen, Germany) with a 32-channel phased array cardiac coil. A balanced steady-state free precession (bSSFP) cine images were acquired covering the entire LV, continuously from the base to the apex on short-axis views, and long-axis views (2-, 3- and 4-chamber) by breath-holding and electrocardiographic gating. The scan parameters were as follows: field of view, 320–340 mm^2^; repetition time, 3.4 ms; echo time, 1.3 ms; slice thickness, 8 mm with no gap; flip angle, 50°; acquisition matrix, 256 × 144; temporal resolution, 42 ms; spatial resolution, 1.4 × 1.3 mm^2^; and number of frames, 25 per cardiac cycle. A phase-sensitive inversion recovery sequence was used to obtain LGE images, 10–15 min after gadolinium (Magnevist; Bayer Healthcare, Berlin, Germany) injection with 0.15 mmol/kg per bolus. The scan parameters were as follows: field of view, 260 × 340 mm^2^; repetition time, 700 ms; echo time, 2.0 ms; delay time after the inversion pulse, 300–380 ms; slice thickness, 8 mm; flip angle, 20°; and matrix size, 116 × 192.

### Image analysis

Biventricular volume, function, and LV mass were measured by Qmass (version 8.1, Medis, Leiden, Netherlands) according to the standardized protocol of the Society for Cardiovascular Magnetic Resonance post-processing guideline [16]. Ventricular mass and volume were indexed to body surface area. The presence of LGE was evaluated by 2 independent readers who were blinded to clinical data. LA volume and function were calculated using the biplane area-length method by tracing LA endocardial contour exclusion of pulmonary veins and LA appendage.

### RA analysis

RA maximum volume, RA volume prior to atrial contraction, and RA minimum volume were assessed using area-length method in the 4-chamber during various RA phase with the exclusion of RA appendage using Qmass (version 8.1, Medis). The RA total empty fraction, RA passive fraction, and RA active fraction were calculated using corresponding RA volumetric measurements.

**Fig. 1:**
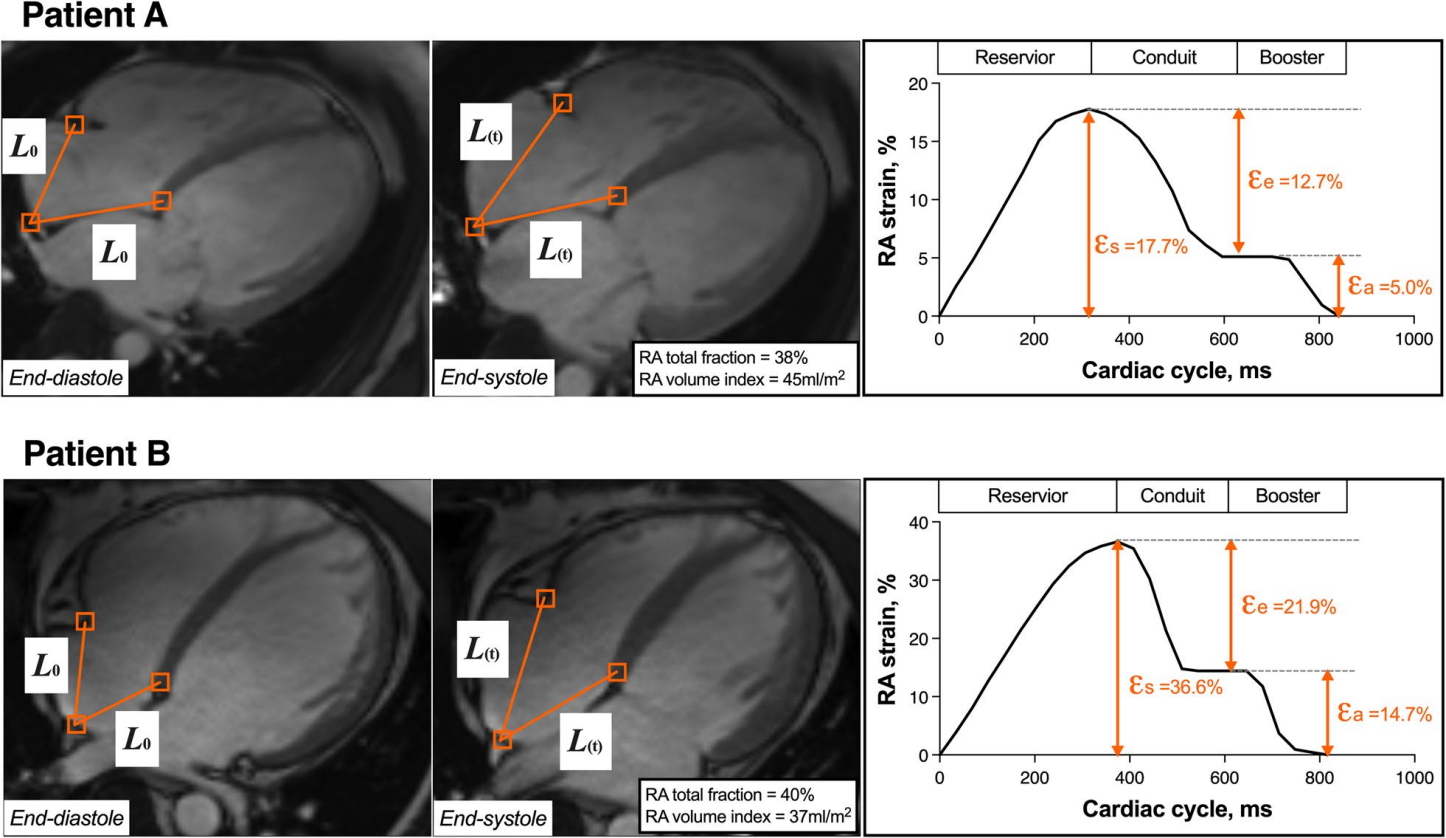
Illustrative examples of measurement of right atrial (RA) strain by fast long-axis method. Three anatomical points were selected at end-diastolic phase and automatically tracking was applied during the cardiac cycle. RA strain was calculated according to the Lagrange strain equation: (L_(t)_ – L_0_) × 100/L_0_. L_(t)_ is the distance between the mid-posterior point and the atrioventricular points at any time (t) during the cardiac cycle. L_0_ is the distance at end-diastole phase. Patient A (upper) and patient B (lower) had similar RA empty fraction but with great difference in strain values, and they presented totally different prognosis. Patient A experienced heart failure death while patient B is still alive. ε_s_: reservoir strain; ε_e_: conduit strain; ε_a_: booster strain

The fast long-axis strain was performed by semi-automated tracking of the length between the mid-posterior RA wall and right atrioventricular junctions on CMR 4-chamber view. The mid-posterior RA wall was selected at the intersection point of RA posterior wall and the RA long-axis, and the right atrioventricular junctions were defined as the tricuspid valve insertion points at the septal and free wall borders of the annuals. The three points were marked at the ventricular end-diastolic phase and tracking was applied through the cardiac cycle using the method of template matching. Manual adjustments were performed when necessary for optimal tracking. RA longitudinal strain then was calculated according to the Lagrange strain equation: (L_(t)_ – L_0_) × 100/L_0_. L_(t)_ is the distance between the mid-posterior point and the atrioventricular points at any time (t) during the cardiac cycle. L_0_ is the distance at RV end-diastole phase. RA reservoir strain, passive strain and active strain were measured at different RA phase. Illustrative example of measurement is shown in Fig. 1.

### Follow-up and clinical outcomes

Patients’ follow-up was conducted until October 2020 by review of medical records, telephone interviews, and contact with the patients’ physician by a cardiologist blinded to CMR data. The cause of hospitalization and death was carefully analyzed according to patients’ symptoms, signs and medical documents. The predefined primary endpoint was all-cause mortality. The composite heart failure (HF) endpoint included HF death, HF readmission, and heart transplantation. The follow-up duration was calculated from the CMR date until endpoint occurred or last contact. Only the first event for patients was included in analysis for composite HF endpoint. Patient data were censored at the time of last follow-up for patients without predefined events.

### Statistical analysis

Normally distributed data were presented as mean ± standard deviation and compared with independent *t* tests, and non-normally distributed data were reported as median (interquartile range) and compared with Mann–Whitney *U* test. Chi-square test or Fisher’s exact test were performed to compare categorical variables as appropriate. Pearson’s and Spearman’s correlation coefficients were used to determine the correlation between RA strain parameters and other clinical or CMR variables, as appropriate. N-terminal pro-brain natriuretic peptide (NT-ProBNP) was analyzed using log transformed values. Cox proportional hazards model was performed to examine the prognostic value of variables in predicting the clinical endpoints. We included variables with *P* < 0.05 in the univariable Cox analysis as covariates in the multivariable model to determine the independent predictors of clinical endpoints. Variance inflation factor (VIF) was calculated to avoid collinearity. One variable was incorporated into the multivariable analysis among parameters with VIF > 3. Survival curves were drawn by Kaplan–Meier method and compared by log-rank test. The incremental prognostic value of RA strain was evaluated by comparing the improvement of Chi-square value of Cox analysis after adding the strain parameters to baseline model. The baseline model included variables with *P* < 0.05 in the univariable Cox analysis. Models were compared by using likelihood ratio test. Receiver operating characteristic (ROC) curves were used to assess the discriminating power between patients with and without clinical endpoints for LA function and volume parameters. The statistical comparison of ROC curves was computed by using the nonparametric DeLong test.

For reproducibility analysis, we randomly selected 20 patients. Interobserver reproducibility was measured by two independent operators blinded to the clinical data. Intraobserver reproducibility was performed by a single operator 2 months later blinded to the results from the first measurements. Then, intraclass correlation coefficient (ICC) and Bland–Altman analysis were performed.

All statistical analysis with two sides and *P* < 0.05 indicated statistical significance. The data were analyzed using SPSS (version 25, Statistical Package for the Social Sciences, International Business Machines, Inc., Armonk, New York, USA) and R (version 4.0.4, The R Foundation for Statistical Computing, Vienna, Austria).

### Results

## Baseline characteristics

This study included 688 patients who met the definition of non-ischemic DCM. Additional 64 patients were excluded from this study due to poor image quality (n = 10), persistent atrial fibrillation (n = 29), and lost-to follow up (n = 25). Thus, the final cohort included 624 patients (Additional file 1: Fig. S1) with a mean age of 48 years, and 444 (71.2%) were men. Table 1 shows the baseline clinical and CMR characteristics of DCM and healthy control participants. No significant difference in sex, age, and body mass index (BMI) was shown between these two groups. Compared with the healthy group, patients with DCM had lower LV ejection fraction (LVEF), RV ejection fraction (RVEF), RA empty fraction, and larger LV and RA volumes (all *P* < 0.001). All three RA phase strain values (reservoir strain, conduit strain, and booster strain) were significantly lower in the DCM cohort (all *P* < 0.001).

**Table 1:** Baseline characteristics of dilated cardiomyopathy patients and healthy volunteers

Parameters	Total patients (n = 624)	Healthy volunteers (n = 120)	*P* value
Age (years)	48 ± 15	48 ± 15	.933
Men, n (%)	444 (71.2)	87 (72.5)	.765
BMI (kg/m^2^)	24.1 ± 8.4	23.0 ± 2.6	.143
LVEDVI (ml/m^2^)	181.2 ± 58.6	75.0 ± 11.4	**< 0.001**
LVESVI (ml/m^2^)	139.0 ± 58.3	26.3 ± 7.0	**< 0.001**
LVEF (%)	25.4 ± 11.7	65.6 ± 6.7	**< 0.001**
LVMI (g/m^2^)	87.3 ± 28.1	50.8 ± 8.7	**< 0.001**
RVEF (%)	36.7 ± 14.6	59.5 ± 6.2	**< 0.001**
LVMI (g/m^2^)	87.3 ± 28.1	50.8 ± 8.7	**< 0.001**
LGE present, n (%)	261 (41.8)	0 (0.0)	**< 0.001**
RAVI max (ml/m^2^)	50.4 ± 23.8	41.5 ± 10.6	**< 0.001**
RAVI p-ac (ml/m^2^)	42.6 ± 22.2	32.1 ± 9.5	**< 0.001**
RAVI min (ml/m^2^)	34.0 ± 22.0	21.4 ± 7.2	**< 0.001**
RA total fraction (%)	35.6 ± 14.0	48.5 ± 8.8	**< 0.001**
RA passive fraction (%)	16.6 ± 7.8	22.2 ± 8.2	**< 0.001**
RA active fraction (%)	23.4 ± 12.2	34.2 ± 8.9	**< 0.001**
RA reservoir strain (%)	23.2 ± 7.2	40.7 ± 10.3	**< 0.001**
RA conduit strain (%)	10.8 ± 6.4	22.1 ± 8.3	**< 0.001**
RA booster strain (%)	12.4 ± 7.2	18.6 ± 4.7	**< 0.001**

BMI: body mass index; LVEDVI: left ventricular end-diastolic volume index; LVESVI: left ventricular end-systolic volume index; LVEF: left ventricular ejection fraction; RVEF: right ventricular ejection function; LVMI: left ventricular mass index; LGE: late gadolinium enhancement; RAVI max: maximal right atrial volume index; RAVI p-ac: right atrial volume index prior to atrial contraction; RAVI min: minimal right atrial volume index

After a median follow-up of 32.5 months (interquartile range: 21.0–47.8 months), 116 patients reached primary endpoint of all-cause mortality, including HF death in 70 patients, sudden cardiac death in 38 patients, and non-cardiac death in 8 patients. A composite of HF secondary endpoints occurred in 205 patients (53 for HF death, 140 for HF readmission, and 12 for heart transplantation). Patients with all-cause mortality were characterized by significantly older and greater New York Heart Association (NYHA) class, serum creatinine, troponin T, NT-ProBNP, LV volume, LA volume, and RA volume; lower BMI, systolic and diastolic blood pressure (BP), LVEF, RVEF, and LA emptying fraction. They had higher prevalence of left bundle branch block (LBBB) and LGE, and were more often treated with diuretics, digoxin, and warfarin. Overall volumetric and strain-based indices of RA function were more impaired in patients who reached primary endpoint (all *P* < 0.001). The results are shown in Table 2.

**Table 2:** Comparison of baseline characteristics between patients with and without death

Parameters	Total patients (n = 624)	Death (n = 116)	Survival (n = 508)	*P* value
Clinical characteristics				
Age (years)	48 ± 15	51 ± 17	47 ± 14	**0.033**
Men, n (%)	444 (71.2)	77 (66.4)	367 (72.2)	0.208
BMI (kg/m^2^)	24.1 ± 8.4	22.4 ± 3.8	24.5 ± 8.7	**0.011**
NYHA class				**< 0.001**
I, n (%)	39 (6.3)	2 (1.7)	37 (7.3)	
II, n (%)	218 (34.9)	27 (23.3)	191 (37.6)	
III, n (%)	265 (42.5)	62 (53.4)	203 (40.0)	
IV, n (%)	102 (16.3)	25 (21.6)	77 (15.2)	
Systolic BP (mmHg)	117 ± 18	111 ± 15	118 ± 19	**< 0.001**
Diastolic BP (mmHg)	76 ± 14	72 ± 11	77 ± 14	**< 0.001**
Heart rates (beats/min)	81 ± 17	80 ± 15	81 ± 17	0.368
Comorbidities				
Smoking, n (%)	292 (46.8)	57 (49.1)	235 (46.3)	0.575
Alcohol, n (%)	186 (29.8)	35 (30.2)	151 (29.7)	0.924
LBBB, n (%)	82 (13.1)	21 (18.1)	61 (12.0)	**0.008**
Hypertension, n (%)	150 (24.0)	22 (19.0)	128 (25.2)	0.156
Diabetes, n (%)	75 (12.0)	19 (16.4)	56 (11.0)	0.109
Laboratory data				
Serum creatinine (umol/L)	80 (69–97)	86 (72–108)	79 (67–94)	**< 0.001**
Troponin T (ng/L)	19 (11–32)	31 (16–52)	17 (10–27)	**< 0.001**
NT-proBNP (pg/ml)	1752 (623–3573)	2957 (1800–6082)	1348 (489–3013)	**< 0.001**
Medications				
ACEI/ARB/ARNI, n (%)	517 (82.9)	91 (78.4)	426 (83.9)	0.163
β-blockers, n (%)	535 (85.7)	95 (81.9)	440 (86.6)	0.190
Spironolactone, n (%)	493 (79.0)	95 (81.9)	398 (78.3)	0.397
Diuretics, n (%)	468 (75.0)	99 (85.3)	369 (72.6)	**0.004**
Digoxin, n (%)	174 (27.9)	49 (42.2)	125 (24.6)	**< 0.001**
Warfarin, n (%)	77 (12.3)	22 (19.0)	55 (10.8)	**0.016**
CMR parameters				
LVEF (%)	25.4 ± 11.7	19.6 ± 7.4	26.6 ± 12.0	**< 0.001**
LVEDVI (ml/m^2^)	181.2 ± 58.6	216.4 ± 59.5	173.7 ± 55.6	**< 0.001**
LVESVI (ml/m^2^)	139.0 ± 58.3	175.8 ± 56.6	131.2 ± 55.7	**< 0.001**
LVMI (g/m^2^)	87.3 ± 28.1	91.8 ± 30.3	86.4 ± 27.6	0.080
RVEF (%)	36.7 ± 14.6	31.8 ± 14.8	37.7 ± 14.3	**< 0.001**
LGE present, n (%)	261 (41.8)	71 (61.2)	191 (37.6)	**< 0.001**
LAEDVI (ml/m^2^)	119.5 ± 65.5	149.6 ± 81.3	112.8 ± 59.5	**< 0.001**
LAEF (%)	34.3 ± 15.8	26.0 ± 11.8	36.1 ± 16.0	**< 0.001**
RAVI max (ml/m^2^)	50.4 ± 23.8	58.3 ± 30.0	48.6 ± 21.8	**0.001**
RAVI p-ac (ml/m^2^)	42.6 ± 22.2	51.0 ± 28.5	40.7 ± 20.1	**< 0.001**
RAVI min (ml/m^2^)	34.0 ± 22.0	43.1 ± 28.4	31.9 ± 19.8	**< 0.001**
RA total fraction (%)	35.6 ± 14.0	30.3 ± 13.8	36.8 ± 13.8	**< 0.001**
RA passive fraction (%)	16.6 ± 7.8	14.3 ± 6.5	17.1 ± 7.9	**0.001**
RA active fraction (%)	23.4 ± 12.2	19.2 ± 11.9	24.3 ± 12.1	**< 0.001**
RA reservoir strain (%)	23.2 ± 7.2	16.5 ± 10.5	24.7 ± 12.2	**< 0.001**
RA conduit strain (%)	10.8 ± 6.4	7.5 ± 4.9	11.5 ± 6.5	**< 0.001**
RA booster strain (%)	12.4 ± 7.2	9.0 ± 6.5	13.2 ± 7.1	**< 0.001**

NYHA class: New York Heart Association class; BP: blood pressure; LBBB: left bundle branch block; NT-proBNP: N-terminal prohormone of brain natriuretic peptide; ACEI: angiotensin-converting enzyme inhibitor; ARB: Angiotensin Receptor Blocker; ARNI: angiotensin receptor neprilysin inhibitor; LAEDVI: left atrial end-diastolic volume index; LAEF: left atrial ejection fraction; other abbreviations as in Table 1

### Survival analysis

**Fig. 2:**
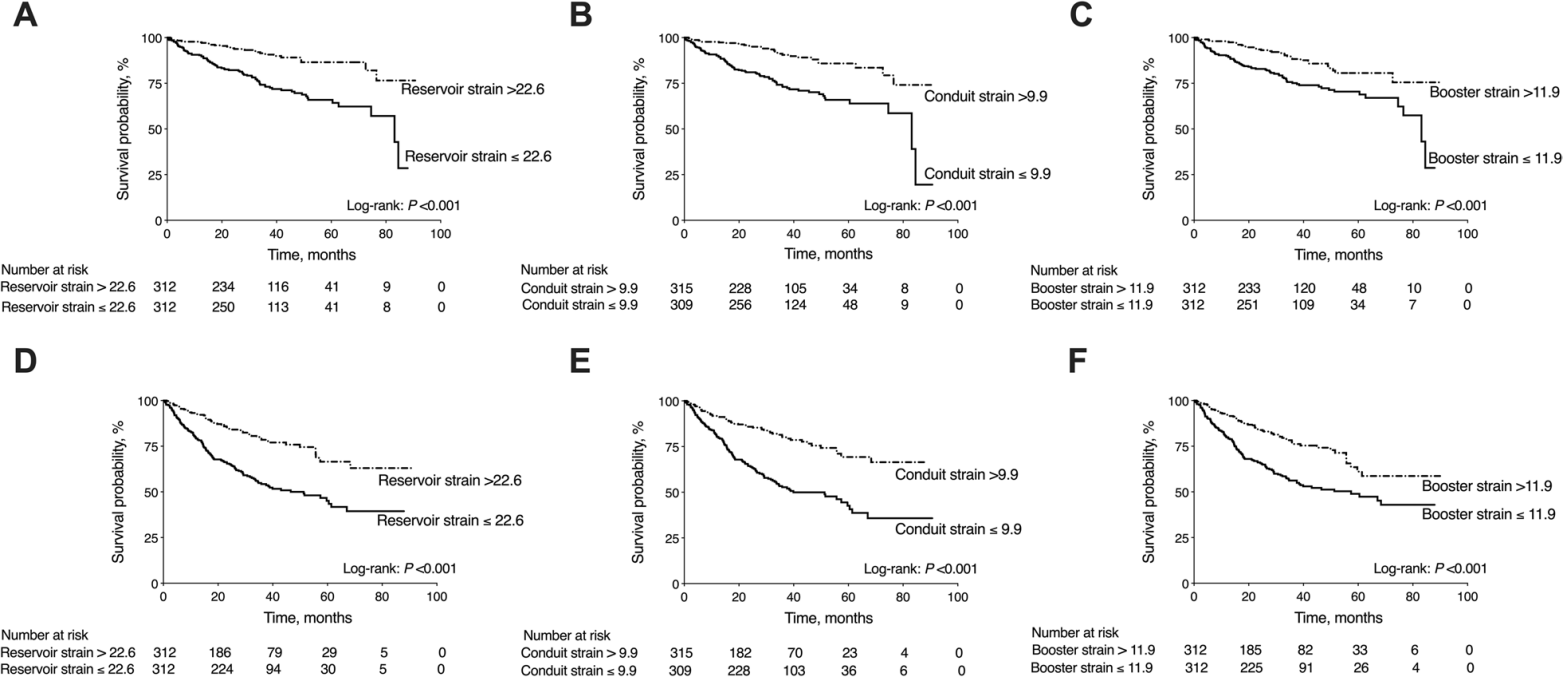
Kaplan–Meier curves of right atrial (RA) strain on clinical outcomes. Patients with RA reservoir strain greater than 22.6, conduit strain greater than 9.9, and booster strain greater than 11.9 experienced significantly lower risk of all-cause mortality (**A**–**C**) and composite heart failure (HF) endpoint (**D**–**F**)

**Fig. 3:**
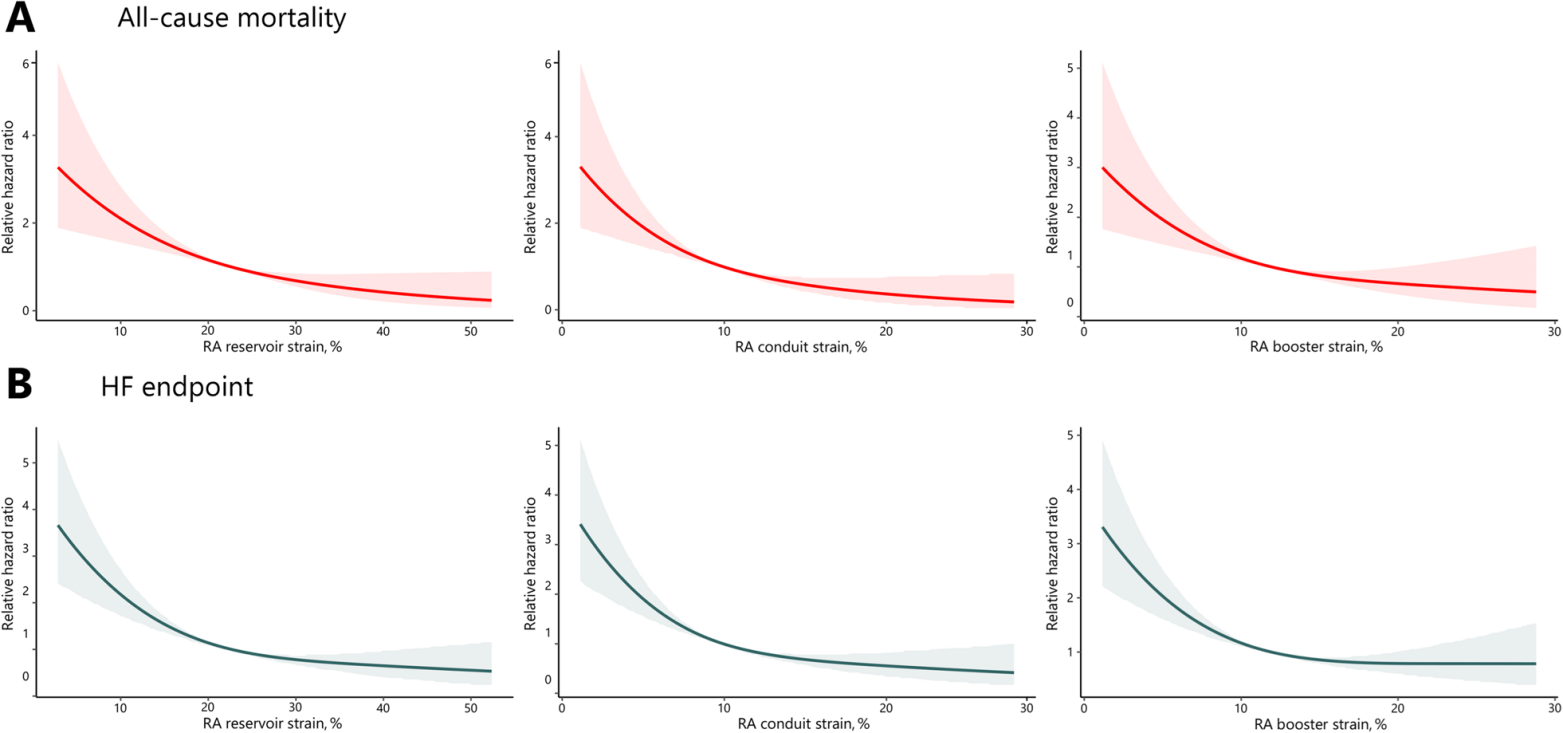
Relationship between right atrial (RA) strain and relative hazard ratio of clinical outcomes (with 95% confidence intervals). Decreased RA reservoir strain, conduit strain, and booster strain showed an increase of hazard ratio of all-cause mortality (**A**) and composite heart failure (HF) endpoint (**B**)

**Fig. 4:**
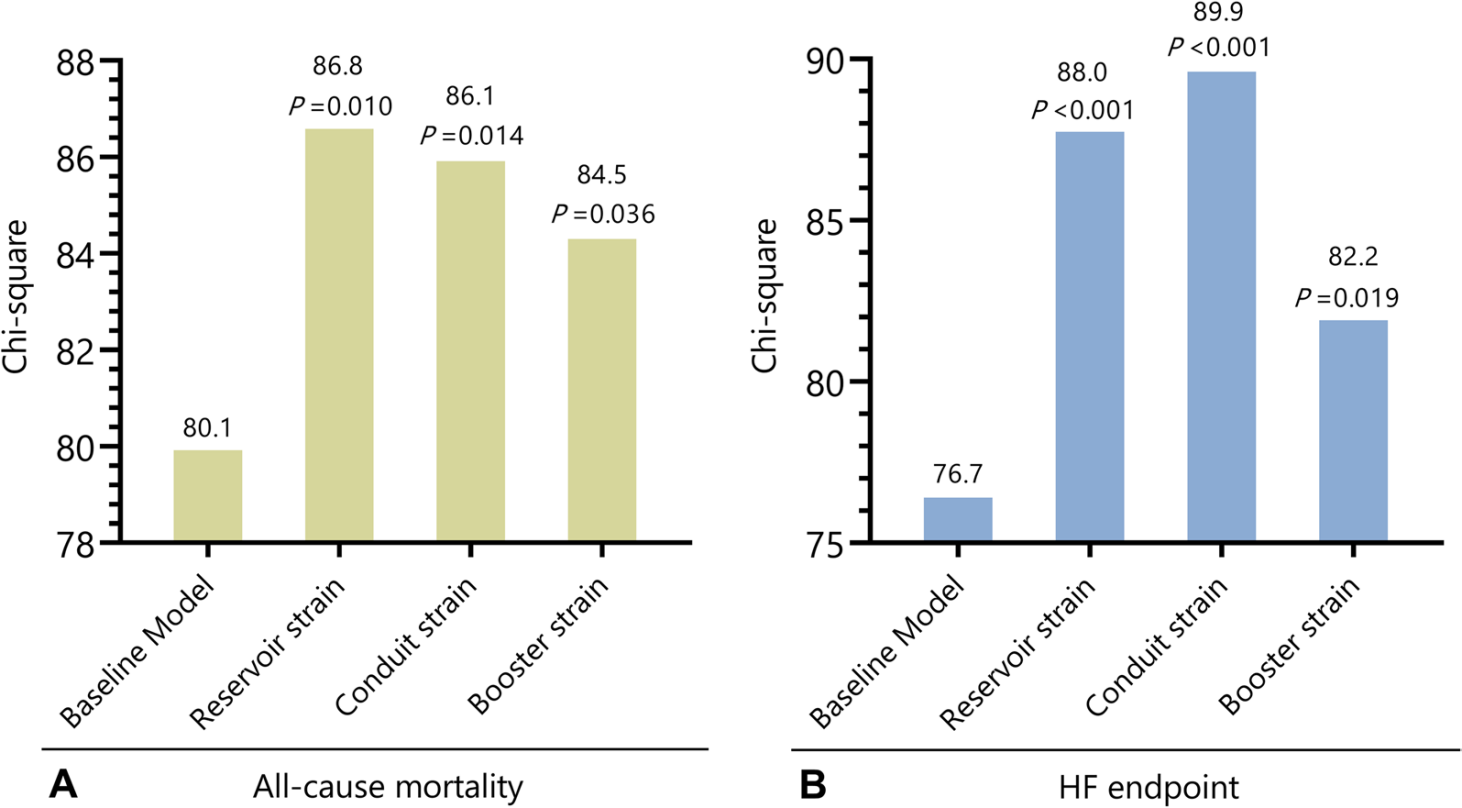
Incremental Value of right atrial (RA) strain over baseline model. RA reservoir strain, conduit strain, and booster strain added incremental prognostic value over clinical and cardiovascular magnetic resonance (CMR) factors for the prediction of all-cause mortality (**A**) and composite heart failure (HF) endpoint (**B**) with a significant improvement of Chi-square value. The baseline model included age, systolic blood pressure, New York Heart Association (NYHA) class, Log (N-terminal prohormone of brain natriuretic peptide), left ventricular (LV) ejection fraction (LVEF), LV end diastolic volume index, right ventricular (RV) ejection fraction (LVEF), left atrial (LA) end diastolic volume index, and late gadolinium enhancement (LGE)

The results of univariable Cox regression analysis are summarized in the Additional file 1: Table S1. All three RA strain parameters were predictors of the all-cause mortality and composite HF endpoint. We incorporated the following covariates in the multivariable regression model: age, systolic BP, NYHA class, Log transformed NT-ProBNP, LVEF, LV end-diastolic volume index, RVEF, LGE, LA end-diastolic volume index, RA minimal volume index, and RA strain. The results showed that RA reservoir strain [hazard ratio (HR) (per 5% decrease) 1.19, 95% confidence interval (CI) 1.03–1.37, *P* = 0.022] and conduit strain [HR (per 5% decrease) 1.37, 95% CI 1.03–1.84, *P* = 0.033] were independent predictors of all-cause mortality, while RA booster strain was not statistically significant in multivariable analysis. Likewise, RA reservoir strain [HR (per 5% decrease) 1.16, 95% CI 1.04–1.30, *P* = 0.008] and RA conduit strain [HR (per 5% decrease) 1.40, 95% CI 1.13–1.74, *P* = 0.002] were independently associated with composite HF endpoint. The results are shown in Tables 3 and 4. Kaplan–Meier analysis stratified by median of RA strain values demonstrated that patients with RA strain better than median (RA reservoir strain > 22.6%, RA conduit strain > 9.9%, and RA booster strain > 11.9%) presented significant lower risk of all-cause mortality and composite HF endpoint than those with RA strain worse than median (All Log rank *P* < 0.001). Results are shown in Fig. 2. The relationship between RA strain and relative hazard ratio is shown in Fig. 3. The cubic splines presented that mortality and HF endpoint risk increased with the decrease of RA strain value. Adding RA strain value to model with clinical and CMR risk factors resulted in a significant increase of chi-square value in the prediction of adverse clinical outcomes (all *P* < 0.05). The results are shown in Fig. 4.

**Table 3:** Multivariate Cox analysis for the prediction of all-cause mortality

	Model 1		Model 2		Model 3	
	HR (95% CI)	*P* value	HR (95% CI)	*P* value	HR (95% CI)	*P* value
Age	1.01 (1.00–1.03)	0.207	1.01 (0.99–1.02)	0.309	1.01 (1.00–1.03)	0.136
Systolic BP	0.99 (0.97–1.00)	0.052	0.99 (0.97–1.00)	**0.046**	0.99 (0.98–1.00)	0.063
NYHA class	0.98 (0.71–1.34)	0.885	1.01 (0.74–1.38)	0.953	0.98 (0.72–1.34)	0.893
Log (NT-proBNP)	2.25 (1.31–3.85)	**0.003**	2.32 (1.36–3.95)	**0.002**	2.36 (1.38–4.04)	**0.002**
LVEF	0.99 (0.96–1.02)	0.504	0.99 (0.96–1.02)	0.553	0.99 (0.95–1.02)	0.359
LVEDVi	1.01 (1.00–1.01)	**0.013**	1.01 (1.00–1.01)	**0.018**	1.01 (1.00–1.01)	**0.018**
RVEF	1.01 (0.99–1.03)	0.270	1.01 (0.99–1.03)	0.302	1.01 (0.99–1.03)	0.391
LGE present	1.68 (1.09–2.59)	**0.020**	1.65 (1.07–2.54)	**0.025**	1.70 (1.10–2.62)	**0.017**
LAEDVI	1.00 (0.99–1.00)	0.514	1.00 (0.99–1.00)	0.431	1.00 (0.99–1.00)	0.478
RAVI min	1.00 (0.99–1.01)	0.975	1.00 (0.99–1.01)	0.734	1.00 (0.99–1.01)	0.786
RA reservoir strain (per 5% decrease)	1.19 (1.03–1.37)	**0.022**	…	…	…	…
RA conduit strain (per 5% decrease)	…	…	1.37 (1.03–1.84)	**0.033**	…	…
RA booster strain (per 5% decrease)	…	…	…	…	1.22 (0.97–1.52)	0.084

Abbreviations as in Table 1 and Table 2;

Model 1 indicates multivariate analysis with RA reservoir strain

Model 2 indicates multivariate analysis with RA conduit strain

Model 3 indicates multivariate analysis with RA booster strain

**Table 4:** Multivariate Cox analysis for the prediction of composite heart failure endpoint

	Model 1		Model 2		Model 3	
	HR (95% CI)	*P* value	HR (95% CI)	*P* value	HR (95% CI)	*P* value
Age	1.01 (1.00–1.02)	0.202	1.01 (0.99–1.02)	0.365	1.01 (1.00–1.02)	0.113
Systolic BP	0.99 (0.98–1.00)	**0.015**	0.99 (0.98–1.00)	**0.010**	0.99 (0.98–1.00)	**0.022**
NYHA class	1.33 (1.05–1.68)	**0.016**	1.36 (1.08–1.71)	**0.009**	1.35 (1.07–1.70)	**0.012**
Log (NT-proBNP)	1.18 (0.82–1.72)	0.374	1.19 (0.82–1.72)	0.361	1.25 (0.86–1.81)	0.241
LVEF	0.99 (0.96–1.01)	0.234	0.99 (0.96–1.01)	0.309	0.98 (0.96–1.01)	0.126
LVEDVI	1.00 (1.00–1.01)	**0.039**	1.00 (1.00–1.01)	**0.045**	1.00 (1.00–1.01)	0.075
RVEF	1.02 (1.00–1.03)	0.067	1.01 (1.00–1.03)	0.056	1.01 (0.99–1.03)	0.168
LGE present	1.26 (0.92–1.73)	0.157	1.23 (0.89–1.69)	0.206	1.29 (0.94–1.77)	0.118
LAEDVI	1.00 (1.00–1.01)	0.353	1.00 (1.00–1.01)	0.308	1.00 (1.00–1.01)	0.307
RAVI min	1.00 (0.99–1.01)	0.493	1.00 (0.99–1.01)	0.309	1.00 (1.00–1.01)	0.207
RA reservoir strain (per 5% decrease)	1.16 (1.04–1.30)	**0.008**	…	…	…	…
RA conduit strain (per 5% decrease)	…	…	1.40 (1.13–1.74)	**0.002**	…	…
RA booster strain (per 5% decrease)	…	…	…	…	1.14 (0.97–1.34)	0.121

Abbreviations as in Table 1 and Table 2;

Model 1 indicates multivariate analysis with RA reservoir strain

Model 2 indicates multivariate analysis with RA conduit strain

Model 3 indicates multivariate analysis with RA booster strain

**Fig. 5:**
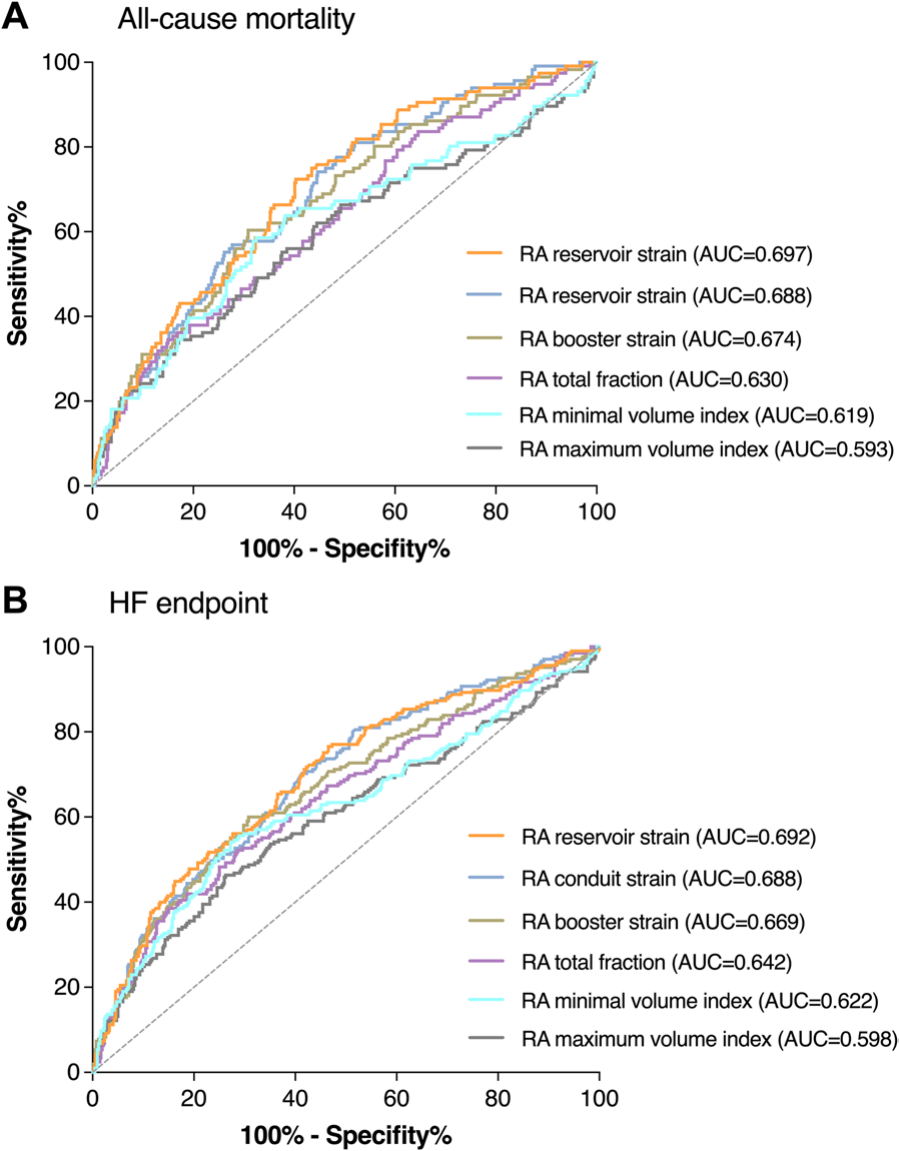
Receiver operating characteristics analysis of right atrial (RA) parameters for the prediction of clinical outcomes. RA strain parameters presented greater area under the curve (AUC) than RA volume and empty fraction for the prediction of all-cause mortality (**A**) and composite heart failure (HF) endpoint (**B**)

ROC curve analysis showed that RA reservoir and conduit strain demonstrated significantly greater area under the curve (AUC) than RA volume and empty fraction for predicting the all-cause mortality and composite HF endpoint (all *P* < 0.05). RA booster strain had similar AUC compared with the RA empty fraction in predicting the adverse events (*P* = 0.06 for all-cause mortality, and *P* = 0.17 for HF endpoint), but still greater than RA volume parameters (*P* < 0.05) (Fig. 5).

In a subgroup analysis among patients with LVEF < 35%, RA reservoir strain and conduit strain remained independent predictors of all-cause mortality and composite HF endpoint (Additional file 1: Table S2).

### Association of RA strain and other clinical and CMR data

RA strains were moderately correlated with RA volume and empty fraction (r = 0.35 to 0.66, all *P* < 0.001), LVEF (r = 0.41 to 0.56, all *P* < 0.001), RVEF (r = 0.54 to 0.62, all *P* < 0.001), LA emptying fraction (r = 0.61 to 0.70, all *P* < 0.001), and reversely correlated with LV end-diastolic volume index (r = − 0.25 to − 0.30, all *P* < 0.001) and Log (NT-ProBNP) (r = − 0.43 to − 0.51, all *P* < 0.001). The results are shown in Table 5.

**Table 5:** Correlation between right atrial strain and other parameters

parameters	RA Reservoir strain		RA Conduit strain		RA Booster strain	
	r value	P value	r value	P value	r value	P value
Log(NT-ProBNP)	− 0.51	**< 0.001**	− 0.50	**< 0.001**	− 0.43	**< 0.001**
LVEF	0.53	**< 0.001**	0.56	**< 0.001**	0.41	**< 0.001**
LVEDVI	− 0.30	**< 0.001**	− 0.29	** < 0.001**	− 0.25	**< 0.001**
RVEF	0.62	**< 0.001**	0.54	**< 0.001**	0.57	**< 0.001**
LAEF	0.70	**< 0.001**	0.66	**< 0.001**	0.61	**< 0.001**
RAVI max	− 0.48	**< 0.001**	− 0.53	**< 0.001**	− 0.59	**< 0.001**
RAVI p-ac	− 0.36	**< 0.001**	− 0.43	**< 0.001**	− 0.46	**< 0.001**
RAVI min	− 0.50	**< 0.001**	− 0.53	**< 0.001**	− 0.60	**< 0.001**
RA total fraction	0.65	**< 0.001**	0.54	**< 0.001**	0.64	**< 0.001**
RA passive fraction	0.50	**< 0.001**	0.57	**< 0.001**	0.35	**< 0.001**
RA active fraction	0.60	**< 0.001**	0.40	**< 0.001**	0.66	**< 0.001**

Abbreviations as in Tables 1 and 2

### Reproducibility analysis

Reproducibility results are presented in Table 6. The intra-observer and inter-observer reproducibility was excellent for RA strain derived from fast long-axis method with all ICC greater than 0.90 and relatively low bias.

**Table 6:** Intra- and interobserver reproducibility for right atrial strain

	Intraobserver		Interobserver	
	ICC	Bias (Limits of agreement)	ICC	Bias (Limits of agreement)
RA reservoir strain	0.98 (0.96–0.99)	− 0.16 (− 3.56 to 3.24)	0.95 (0.89–0.98)	− 1.63 (− 7.47 to 4.21)
RA conduit strain	0.96 (0.91–0.99)	− 0.03 (− 2.76 to 2.76)	0.92 (0.81–0.97)	− 1.42 (− 5.73 to 2.90)
RA booster strain	0.98 (0.96–0.99)	− 0.14 (− 2.42 to 2.14)	0.93 (0.84–0.97)	− 0.29 (− 4.91 to 4.34)

ICC: intraclass correlation coefficient; RA: right atrial

## Discussion

In this study, we explored the prognostic value of RA strain derived using fast long-axis method in DCM patients. The main findings are: (1) DCM patients had impaired RA empty function and strain compared with sex- and age-matched healthy controls; (2) The RA strain was independently associated with primary endpoint of all-cause mortality and composite HF endpoint of HF readmission, HF death, and heart transplantation. Patients with RA strain values lower than median strain values experienced significantly higher risk of adverse clinical endpoints. (3) RA strain added incremental prognostic value over traditional clinical and CMR risk factors; (4) RA strain was correlated with RA volumetric derived empty function, LV and RV function, and Log (NT-ProBNP).

The recognition of RA function beyond a “receptacle and store-house” as it was first described [17] has improved recently. The RA plays an important role in modulating the interactions between the performance of different chambers and systemic circulation. The normal mechanical function of RA provides sufficient return of blood to the heart, avoids venous congestion, and protects the upstreaming organs [8]. The sinoatrial node severs as the generation of cardiac impulse and the endocrine function is pivotal in volume regulation in response to acute myocytes stretch and neurohumoral activation [18]. Previous studies showed RA structural and functional remodeling are prevalent in patients with HF, pulmonary artery hypertension, and hypertrophic cardiomyopathy [19–21]. Tigen et al*.* found worse RA function compared to healthy subjects in a small non-ischemic DCM cohort using two-dimensional speckle tracking echocardiography [22]. Our study further demonstrated that RA volume, empty fraction, and strain values were greatly impaired in DCM patients. The prominent ventricular overloading, systolic and diastolic dysfunction in DCM patients inevitably influenced atrial performance. The intrinsic atrial characteristics alterations such as atrial fibrosis and atrial myopathy may develop in DCM patients. These factors contributed to fRA enlargement and dysfunction.

RA structural and functional remodeling has been reported as the risk predictors in limited studies. Sallach et al*.* reported that RA volume index was an independent predictor of death, transplantation, and HF readmission in patients with chronic systolic HF [23]. Then Jain et al*.* described the patterns of RA dysfunction in HF with preserved and reduced ejection fraction and found that RA reservoir and conduit function were independently associated with death. However, the prognostic analysis was conducted in a diverse group including patients with and without HF [19]. Another study demonstrated that RA conduit function precisely identified HF degree and was an independent risk factor in patients with acute myocardial infarction [24]. In DCM, D’Andrea et al*.* found that the more increased RA area index and impaired RA myocardial deformation measured by two-dimensional strain echocardiography indicated non-responders to cardiac resynchronization therapy in DCM patients, suggesting the potentially poor prognosis in DCM with severely impaired RA function [25].

Our study explored the prognostic value of RA strain in patients with DCM and found that RA strain functions were independent predictors of adverse clinical events. Moreover, impaired RA function demonstrated significant additional prognostic value over traditional clinical and CMR risk factors. Since atrial enlargement can occur in an asymmetrical way, the fact that we only studied the 4-chamber view may affect the accuracy of RA volume and corresponding empty fraction measurement [15]. Our fast long-axis strain only tracked three anatomical prominent points to assess the RA longitudinal deformation. The results showed that RA strain based on this method was superior to RA volume and volume-derived empty fraction in the prediction of adverse clinical endpoints.

The pathophysiological relationship between RA strain function and clinical endpoints is not fully understood. In DCM, LV systolic and diastolic dysfunction associated with elevated pulmonary pressure increases RV overload, impairs RV diastolic dysfunction, and increase functional tricuspid regurgitation which progresses to RA failure as a result of Frank-Starling mechanism. The dilated RA chamber and impaired RA function then further contributed to a decrease of the cardiac output. Our study also showed that RA function was correlated with biventricular performance. However, we found that patients with severely decreased LVEF (< 35%) exhibited totally different burdens of symptoms and prognosis, and the RA strain was still independently associated with adverse endpoints among these patients, indicating that RA function may play an important role in the risk stratification in DCM patients, more than just a reflection of decreased left side heart performance. This can be explained by the RA unique functions. First, the RA dysfunction is associated with the development of dyspnea and decompensation in HF patients by the worsening peripheral congestion. Second, the neurohormonal disturbance by the fibrotic stretch of RA manifested as the excessively defective atrial natriuretic peptide synthesis and natriuretic peptide resistance, leading to volume overload and sodium retention [26]. Moreover, RA myocardial alteration and atrial fibrosis may influence the electromechanical properties. Previous studies found that lower RA strain and higher RA volume were associated with incident atrial fibrillation occurrence, which was a factor for poor prognosis [27].

This study has important clinical implication. In the management and treatment of patients with DCM, we mainly focus on the LV function, and RA function has been neglected. Since the risk stratification for DCM patients remains challenging in clinical routine, it should be optimized by more useful clinical variables, imaging techniques and genetic analysis [28]. We provided a fast, reliable, and software-independent way to measure the RA myocardium change during the cardiac cycle. The results showed that RA phasic strain was independent predictor of adverse clinical outcomes and provided incremental prognostic value versus cardiac risk factors. Thus, the interventions targeting at mitigating RA remodeling and improving RA function such as the use of diuretics, alleviation of pulmonary hypertension, and treatment of atrial fibrillation may relieve the patients’ symptoms. The dynamic monitoring of RA function is needed. Besides, to what extent the RA remodeling could be reversed after treatment and its association with clinical outcomes should be explored in the future.

## Limitations

Our study has several limitations. First, this is a single-center study conducted only on Chinese adult, and the application of RA volume and functional parameters might be limited for other groups. Second, in this study, we measured the RA volume only from the 4-chamber view by area-length method rather than short-axis method. Third, patients with persistent atrial fibrillation were excluded from the study, which influenced the generalization of the conclusion. Moreover, the follow-up information on arrhythmia events was not concluded in this study. Future studies are warranted to explore the association between RA function and arrhythmia.

## Conclusions

In conclusion, RA structure and function are impaired in DCM patients. RA phasic strain are independent predictors of all-cause mortality and HF composite endpoints, and provided incremental prognostic value over clinical and CMR parameters. Compared with RA empty fraction and volume, RA strain has higher predictive value.

## Supplementary Information

**Additional file 1: Figure S1. **Patient inclusion flowchart. CMR, cardiovascular magnetic resonance. **Table S1.** Univariable Cox analysis for all-cause mortality and composite heart failure endpoint. **Table S2.** Multivariable analysis of right atrial strain among patients with LVEF < 35%.

## Data Availability

The datasets used and/or analyzed during the current study are available from the corresponding author on reasonable request.

## Abbreviations

BMI: Body mass index
BP: Blood pressure
bSSFP: Balanced steady-state free precession
CMR: Cardiovascular magnetic resonance
DCM: Dilated cardiomyopathy
HF: Heart failure
ICC: Intraclass correlation coefficient
LA: Left atrium/left atrial
LGE: Late gadolinium enhancement
LV: Left ventricle/left ventricular
LVEF: Left ventricular ejection fraction
NT-proBNP: N-terminal pro-brain natriuretic peptide
NYHA: New York Heart Association
RA: Right atrium/right atrial
RAVI: Right atrial volume index
ROC: Receiver operating characteristic
RV: Right ventricle/right ventricular
RVEF: Right ventricular ejection fraction
VIF: Variance inflation factor
