# Supplementary material for: Prognostic value of right atrial strain derived from cardiovascular magnetic resonance in non-ischemic dilated cardiomyopathy
Source: J Cardiovasc Magn Reson. 2022 Nov 10;24:54. doi: 10.1186/s12968-022-00894-w (PMC9648034; doi:10.1186/s12968-022-00894-w)
Supplement: Supplementary file 1 — Additional file 1: Figure S1. Patient inclusion flowchart. CMR, cardiovascular magnetic resonance. Table S1. Univariable Cox analysis for all-cause mortality and composite heart failure endpoint. Table S2. Multivariable analysis of right atrial strain among patients with LVEF < 35%. [file 12968_2022_894_MOESM1_ESM.docx]

**Supplementary materials**

**Figure S1. Patient inclusion flowchart.** CMR, cardiovascular magnetic resonance.


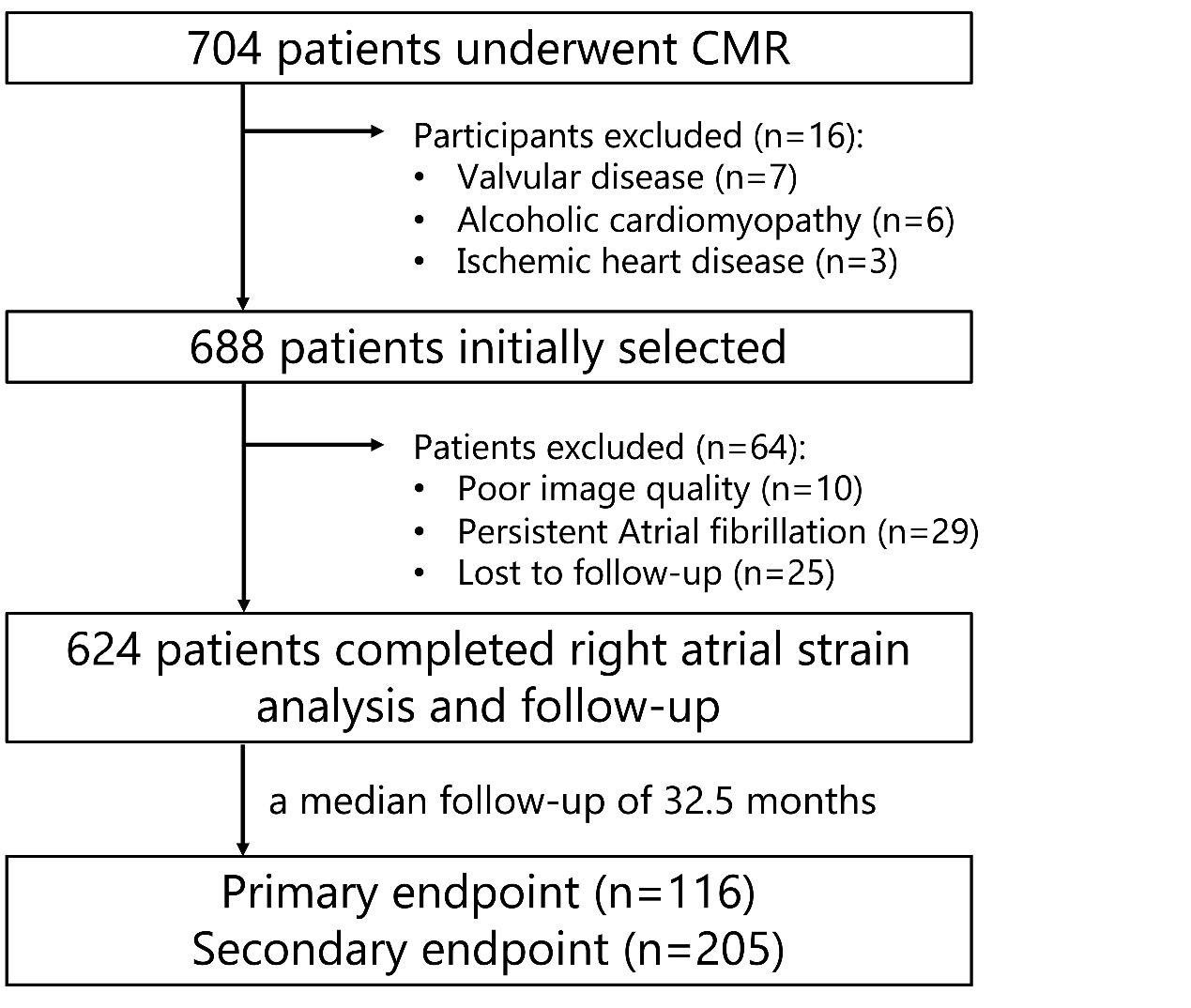


Table S1. Univariable Cox analysis for all-cause mortality and composite heart failure endpoint

| Parameter | All-cause mortality | | Composite HF endpoint | |
| --- | --- | --- | --- | --- |
|  | HR (95% CI) | *P* value | HR (95% CI) | *P* value |
| Age | 1.02 (1.00-1.03) | **0.012** | 1.01 (1.00-1.02) | **0.006** |
| Sex | 1.30 (0.88-1.91) | 0.182 | 1.14 (0.85-1.54) | .0375 |
| NYHA class | 1.60 (1.26-2.02) | **<0.001** | 1.79 (1.50-2.13) | **<0.001** |
| Systolic BP | 0.98 (0.97-0.99) | **<0.001** | 0.99 (0.98-0.99) | **<0.001** |
| Smoking | 1.02 (0.71-1.47) | 0.993 | 1.11 (0.84-1.46) | 0.453 |
| Alcohol | 1.00 (0.67-1.49) | 0.995 | 1.12 (0.91-1.62) | 0.198 |
| LBBB | 0.73 (0.45-1.17) | 0.185 | 0.92 (0.62-1.35) | 0.664 |
| Log (NT-proBNP) | 3.32 (2.24-4.94) | **<0.001** | 2.20 (1.68-2.89) | **<0.001** |
| LVEF | 0.93 (0.91-0.96) | **<0.001** | 0.95 (0.94-0.97) | **<0.001** |
| LVEDVI | 1.01 (1.01-1.02) | **<0.001** | 1.01 (1.00-1.01) | **<0.001** |
| LVESVI | 1.01 (1.01-1.02) | **<0.001** | 1.01 (1.00-1.01) | **<0.001** |
| LVMI | 1.01 (0.99-1.01) | 0.056 | 1.00 (0.99-1.01) | 0.966 |
| RVEF | 0.97 (0.96-0.99) | **<0.001** | 0.98 (0.97-0.99) | **<0.001** |
| LGE present | 2.63 (1.80-3.82) | **<0.001** | 1.96 (1.49-2.58) | **<0.001** |
| LAEDVI | 1.01 (1.00-1.01) | **<0.001** | 1.01 (1.00-1.01) | **<0.001** |
| LAEF | 0.96 (0.94-0.97) | **<0.001** | 0.96 (0.95-0.97) | **<0.001** |
| RAVI max | 1.01 (1.01-1.02) | **<0.001** | 1.01 (1.00-1.01) | **<0.001** |
| RAVI p-ac | 1.01 (1.01-1.02) | **<0.001** | 1.01 (1.01-1.02) | **<0.001** |
| RAIi min | 1.01 (1.01-1.02) | **<0.001** | 1.01 (1.01-1.02) | **<0.001** |
| RA total fraction | 0.97 (0.96-0.99) | **<0.001** | 0.97 (0.96-0.98) | **<0.001** |
| RA passive fraction | 0.96 (0.93-0.98) | **0.001** | 0.96 (0.94-0.98) | **<0.001** |
| RA active fraction | 0.97 (0.96-0.99) | **0.001** | 0.97 (0.96-0.98) | **<0.001** |
| RA reservoir strain (per 5% decrease) | 1.32 (1.21-1.45) | **<0.001** | 1.27 (1.19-1.36) | **<0.001** |
| RA conduit strain (per 5% decrease) | 1.83 (1.51-2.23) | **<0.001** | 1.64 (1.43-1.88) | **<0.001** |
| RA booster strain (per 5% decrease) | 1.48 (1.27-1.72) | **<0.001** | 1.41 (1.26-1.57) | **<0.001** |

NYHA class, New York Heart Association class; BP, blood pressure; LBBB, left bundle branch block; Log (NT-proBNP), log transformed N-terminal prohormone of brain natriuretic peptide; LVEF, left ventricular ejection fraction; LVEDVI, left ventricular end-diastolic volume index; LVESVI, left ventricular end-systolic volume index; LVMI, left ventricular mass index; RVEF, right ventricular ejection function; LGE, late gadolinium enhancement; LAEDVI, left atrial end-diastolic volume index; LAEF, left atrial emtying fraction; RA right atrium.

Table S2. Multivariable analysis of right atrial strain among patients with LVEF<35%.

|  | All-cause mortality | | Composite HF endpoint | |
| --- | --- | --- | --- | --- |
|  | HR (95% CI) | *P* value | HR (95% CI) | *P* value |
| RA reservoir strain (per 5% decrease) | 1.20 (1.02 – 1.40) | **0.026** | 1.16 (1.03 – 1.31) | **0.015** |
| RA conduit strain (per 5% decrease) | 1.45 (1.07 – 1.97) | **0.017** | 1.47 (1.16 – 1.85) | **0.001** |
| RA booster strain (per 5% decrease) | 1.18 (0.93 – 1.51) | 0.176 | 1.09 (0.91 – 1.30) | 0.368 |

Multivariable analysis was adjusted for systolic blood pressure, log transformed N-terminal prohormone of brain natriuretic peptide, New York Heart Association class, left ventricular end-diastolic volume index, right ventricular ejection function, late gadolinium enhancement, right atrial minimal volume index, and right atrial strain parameters.

RA, right atrial; HF, heart failure.
